# Verification of Single Nucleotide Polymorphisms rs34554140, rs6670279, and rs6874185 as Novel Molecular Genetic Markers of Sudden Cardiac Death

**DOI:** 10.17691/stm2021.13.2.04

**Published:** 2021-01-01

**Authors:** A.A. Ivanova, A.A. Gurazheva, E.S. Melnikova, A.M. Nesterets, S.K. Malyutina, I.A. Rodina, V.N. Maksimov

**Affiliations:** Senior Researcher, Laboratory for Molecular Genetics of Internal Diseases, Institution of Internal and Preventive Medicine — Branch of the Federal Research Center Institute of Cytology and Genetics, Siberian Branch of the Russian Academy of Sciences, 175/1 Borisa Bogatkova St., Novosibirsk, 630089, Russia; Junior Researcher, Laboratory for Molecular Genetics of Internal Diseases, Institution of Internal and Preventive Medicine — Branch of the Federal Research Center Institute of Cytology and Genetics, Siberian Branch of the Russian Academy of Sciences, 175/1 Borisa Bogatkova St., Novosibirsk, 630089, Russia; PhD Student, Institution of Internal and Preventive Medicine — Branch of the Federal Research Center Institute of Cytology and Genetics, Siberian Branch of the Russian Academy of Sciences, 175/1 Borisa Bogatkova St., Novosibirsk, 630089, Russia; PhD Student, Institution of Internal and Preventive Medicine — Branch of the Federal Research Center Institute of Cytology and Genetics, Siberian Branch of the Russian Academy of Sciences, 175/1 Borisa Bogatkova St., Novosibirsk, 630089, Russia; Professor, Department of Internal Medicine, Hematology and Transfusiology, Faculty of Advanced Medical Training and Professional Retraining, Novosibirsk State Medical University, 52 Krasny Prospekt, Novosibirsk, 630091, Russia; Head of the Laboratory for Etiopathogenesis of Internal Diseases, Institution of Internal and Preventive Medicine — Branch of the Federal Research Center Institute of Cytology and Genetics, Siberian Branch of the Russian Academy of Sciences, 175/1 Borisa Bogatkova St., Novosibirsk, 630089, Russia; Forensic Expert, Novosibirsk Regional Clinical Bureau of Forensic Medicine, 134 Nemirovich-Danchenko St., Novosibirsk, 630087, Russia; Professor, Head of the Laboratory for Molecular Genetics of Internal Diseases, Institution of Internal and Preventive Medicine — Branch of the Federal Research Center Institute of Cytology and Genetics, Siberian Branch of the Russian Academy of Sciences, 175/1 Borisa Bogatkova St., Novosibirsk, 630089, Russia; Professor, Department of Medical Genetics and Biology, Faculty of Medicine, Novosibirsk State Medical University, 52 Krasny Prospekt, Novosibirsk, 630091, Russia

**Keywords:** sudden cardiac death, single nucleotide polymorphism, rs34554140, rs6670279, rs6874185, molecular genetic marker

## Abstract

**The aim of the study** was to explore the association between sudden cardiac death (SCD) and single nucleotide polymorphisms (SNPs) rs34554140, rs6670279, and rs6874185 from the list of potential molecular genetic markers of SCD, obtained in our earlier genome-wide allelotyping on pooled DNA samples.

**Materials and Methods:**

The study is based on the case-control principle. The SCD group included 438 deceased residents of Novosibirsk (average age — 53.2±9.1 years; men — 72.7%, women — 28.3%) with the main postmortem diagnoses of acute circulatory failure or acute coronary failure, which met the criteria of SCD established by the European Society of Cardiology. The control group included 435 live subjects enrolled in the international projects HAPIEE and MONICA (average age — 53.2±8.9 years; men — 70.0%, women — 30.0%). DNA was isolated by phenol-chloroform extraction from the myocardial tissue in the SCD group and from the venous blood in the control group. Genotyping was performed by polymerase chain reaction with subsequent analysis of restriction fragment length polymorphism in a polyacrylamide gel.

**Results:**

The frequencies of the genotypes of SNPs rs34554140, rs6670279, and rs6874185 in the control group correspond to those predicted by the Hardy–Weinberg equilibrium (c^2^=0.98, 0.009, 3.39, respectively). The AA genotype of rs34554140 is associated with an increased risk of SCD (p=0.002; OR=1.85; 95% CI 1.26–2.71). The AT genotype has a protective effect against SCD (p=0.001; OR=0.53; 95% CI 0.36–0.78). In subgroups separated by gender and age, the differences persist in the subgroups of men, women, and individuals under 50 years old (p<0.05). The AA genotype of rs6670279 is associated with an increased risk of SCD (p=0.005; OR=1.54; 95% CI 1.15–2.06). The AT genotype has a protective effect against SCD (p=0.047; OR=0.73; 95% CI 0.54–0.98). When distributed by sex and age, the differences persist in the subgroups of men, individuals above 50 years old, and men above 50 years old (p<0.05). There were no significant differences in the frequencies of genotypes and alleles of rs6874185 between the SCD and control groups, even after the subgroups specified by gender and age were compared (p>0.05).

**Conclusion:**

The association of single nucleotide polymorphisms rs34554140 and rs6670279 with SCD was confirmed. In contrast, no association of rs6874185 with SCD was detected.

## Introduction

Sudden cardiac death (SCD) continues to be one of the unsolved problems of modern health care. Despite the commonly used preventive measures, SCD remains the leading cause of cardiovascular mortality, which may result from both acquired and hereditary diseases [1]. For patients diagnosed with a cardiac disease, the SCD risk stratification is crucial for patient management [2]. However, in most cases, SCD develops on the background of previously undiagnosed cardiovascular disease, which is detected posthumously. At autopsy, though, it is not always possible to determine the exact causes of SCD; in these cases, the diagnosis of cardiac rhythm disturbance is often put forward [3]. It is now believed that genetic testing should be included in the SCD risk assessment. Therefore, molecular genetic markers of SCD are actively studied worldwide in a hope to find sensitive and predictive ones to be included in SCD risk meters.

In our earlier genome-wide allelotyping analysis of human DNA [4], a number of potential molecular genetic markers of SCD were identified. To rule out false-positive findings, we had to reassess these results by using routine case-control methods. Therefore, the **aim of the present study** was to verify the association of single nucleotide polymorphisms rs34554140, rs6670279, and rs6874185 with sudden cardiac death.

## Materials and Methods

The study design is based on the case-control principle. To form the SCD group, we used an archival anonymous DNA bank containing data on Novosibirsk residents who died from sudden death. The forensic medical examination of these subjects was carried out at the Novosibirsk Regional Clinical Bureau of Forensic Medicine (Russia). In the forensic study, a sample of myocardial tissue weighing 5–10 g was taken from a suddenly deceased person; the sample was subsequently stored (until the stage of DNA extraction) at –20°C. DNA was isolated from the myocardial tissue by phenol-chloroform extraction.

Based on the personal data and the medical records, the SCD group (n=438, average age — 53.2±9.1 years, men — 72.7%, women — 28.3%) consisted of DNA samples of the deceased with pathological (postmortem) diagnoses of acute circulatory failure and/or acute coronary insufficiency (according to the criteria of the European Society of Cardiology, ESC) [3]. Excluded from this group were persons containing alcohol or drugs in their blood and those with morphological signs of myocardial infarction or cardiomyopathies.

For the control group, we used data from the international DNA bank created within the MONICA (Multinational Monitoring of Trends and Determinants in Cardiovascular Disease) and HAPIEE (Health, Alcohol and Psychosocial factors in Eastern Europe) projects. The control group (n=435, average age — 53.2±8.9 years, men — 70.0%, women — 30.0%) matched the SCD group by sex and age and included individuals who were alive at the time when the above projects were running. In these projects, the venous blood of the participants was taken into tubes with EDTA and further stored at a temperature of –20°C until the DNA extraction procedure. The DNA was prepared from venous blood by phenol-chloroform extraction.

Genotyping was performed by polymerase chain reaction (PCR) with subsequent analysis of restriction fragment length polymorphisms according to the author’s protocols.

The primers 5’-CTGGAAGCAGCTAGACAGC-3’(F) and 5’-GGTCAGGGAGACACACTCG-3’(R) were used for genotyping of rs34554140 nucleotide polymorphism. The PCR reagent mixture contained (in a volume of 25 μl) Tris-HCI (pH 9.0) — 75 mmol; (NH_4_)_2_ SO_4_ — 20 mmol; Tween-20 — 0.01%; MgCl_2_ — 2.5 mmol; 0.8 mmol of each primer; dNTP mixture — 0.2 mmol; DNA — 2 μg; 1 unit activity of Taq-DNA polymerase (SibEnzyme, Novosibirsk, Russia). Amplification was carried out as follows: 33 cycles, including denaturation at 95°C (30 s), primer annealing at 58°C (30 s), and elongation at 72°C (30 s). Restriction was carried out using 10 activity units of TaqI restriction enzyme (SibEnzyme). The amplification product had a size of 195 bp. In the AA genotype, a 195 bp product was detected, and in the AT genotype — 195, 176, and 19 bp products, and in the TT genotype — 19, 176 bp products.

The primers 5’-GACACCTGGATGAGCTGCACAGC-3’(F) and 5’-CCCGGCCAAATTTGTGTTG-3’(R) were used for rs6670279 genotyping. The PCR reagent mixture contained (in a volume of 25 μl) Tris-HCI (pH 9.0) — 75 mmol; (NH_4_)_2_ SO_4_ — 20 mmol; Tween-20 — 0.01%; MgCl_2_ — 3.0 mmol; 0.9 mmol of each primer; dNTP mixture — 0.2 mmol; DNA — 2 μg; 1 unit activity of Taq-DNA polymerase (SibEnzyme). Amplification was carried out under the following temperature regime: 35 cycles, including denaturation at 95°C (30 s), annealing of primers at 62°C (30 s), and elongation at 72°C (30 s). Restriction was carried out with 10 activity units of the restriction enzyme PvuII (SibEnzyme). The size of the amplification product was 158 bp. After restriction, in the AA genotype, a product of 158 bp was detected, in the AT genotype, the products were 158, 136, 22 bp, and in the TT genotype, the products were 136, 22 bp.

The primers 5’-AGCAGCTCTTAGCTGTGACT-3’(F) and 5’-ATCTCAGAGCCCTCAAAGC-3’(R) were used for rs6874185 genotyping. The PCR reagent mixture contained (in a volume of 25 μl) Tris-HCI (pH 9.0) — 75 mmol; (NH_4_)_2_ SO_4_ — 20 mmol; Tween-20 — 0.01%; MgCl_2_ — 3.0 mmol; 0.8 mmol of each primer; dNTP mixture — 0.2 mmol; DNA — 2 μg; 1 unit activity of SynTaq-DNA polymerase with the enzyme-inhibiting antibodies (Syntol, Moscow). Amplification was carried out under the following temperature regime: 35 cycles, including denaturation at 95°C (30 s), annealing of primers at 56°C (30 s), and elongation at 72°C (30 s). Restriction was carried out with 10 activity units of the restriction enzyme HinfI (SibEnzyme). The size of the amplification product was 237 bp. After restriction, with the TT genotype, a 237 bp product was detected, with the CT genotype — 237, 220, and 17 bp products, and with the CC genotype — 220 and 17 bp products.

In order to assess the mechanism by which the selected SNPs could be associated with SCD, we analyzed the data characterizing the heart conditions preceding SCD. We also looked at the risk factors for SCD and cardiovascular diseases, which could predict the development of SCD (atherosclerosis, heart mass, thickness of the interventricular septum, myocardium of the right and left ventricles). The analysis also included data from anthropometric, clinical, and biochemical examinations of control subjects (concentration of cholesterol, high and low-density lipoproteins, and triglycerides; atherogenic index; systolic and diastolic blood pressure; pulse pressure; body mass index; waist circumference; fasting plasma glucose; heart rate), which could be associated with the risk of SCD.

The frequencies of genotypes and alleles of these polymorphisms were compared between the case and control groups. Then the groups were subdivided by gender to calculate the respective frequencies of males and females in the case and control groups. Similarly, the subgrouping was made by age (<50 years old and >50 years old), then by both gender and age where the frequencies in the subgroup of women <50 y.o. were compared as case vs control, subgroups of men <50 y.o. — as case vs control, women >50 y.o. as case vs control, and men >50 y.o. as case vs control. In this subgrouping, we aimed to identify the gender and age, at which a specific genotype might be associated with a high or low risk of SCD. Sometimes, in the presence of statistically significant differences in the total group, it turned out that the genotype retained its association with the diagnosis (for example, only men >50 y.o.), which led us to conclude that this polymorphism significantly contributed to the development of the given disease in this age and gender subgroup.

**Statistical processing of the results** was carried out using the SPSS 16.0 software package; the frequencies of genotypes and alleles of the SNPs were determined in the SCD and control groups. Using criterion c^2^, we tested whether the calculated frequencies matched the Hardy–Weinberg equilibrium in the control group. Comparison of the genotype and allele frequencies between the groups was carried out using contingency tables and the Pearson’s c^2^ criterion. With the four-field tables, Fisher’s exact two-sided test with Yates’ correction for continuity was used. The relative risk of SCD was calculated as the odds ratio using Fisher’s exact two-sided test and Pearson’s <a>^2^ criterion. The significance of the differences was confirmed at p<0.05.

The normality of the data distribution in the forensic, clinical, anthropometric, and biochemical studies was tested using the Kolmogorov–Smirnov method. With the normal distribution, calculations were performed using the ANOVA test. To assess the correctness of the chosen calculation method, the Leuven test for dispersion homogeneity was used. The Duncan’s test was used to determine whether the mean values differed between the subgroups. In case of deviation from the normal distribution, the Kruskal–Wallis test and the Mann–Whitney test were used. Data expressed on the nominal and ordinal scales were analyzed using contingency tables and Pearson’s <a>^2^ test, corrected for likelihood. P<0.05 was taken as the level of significance.

The study protocol complied with the ethical standards provided by the Declaration of Helsinki (2013) and the Rules of Good Clinical Practice, approved by the Order of the Ministry of Health of the Russian Federation of April 1, 2016, No.200n. The study was approved by the Ethics Committee of the Research Institute of Therapy and Preventive Medicine affiliated with the Federal Research Center Institute of Cytology and Genetics, Siberian Branch of the Russian Academy of Sciences.

## Results

In the control group, the observed frequencies of genotypes of single nucleotide polymorphisms rs34554140, rs6670279, and rs6874185 were close to those predicted by the Hardy–Weinberg equilibrium (<a>^2^=0.98, 0.009, 3.39).

There were no statistically significant differences between the SCD and control groups regarding the frequencies of genotypes and alleles of rs6874185, even after the groups were subgrouped by gender or age (p>0.05) (see the Figure).

**Figure:**
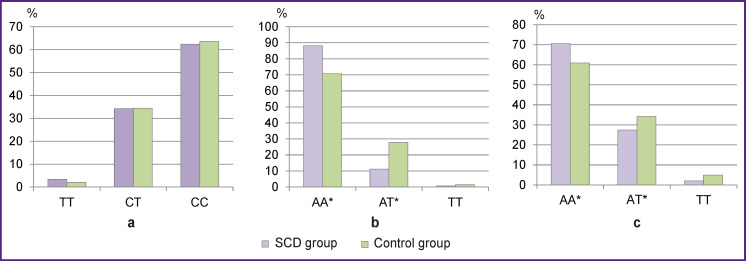
Frequencies of genotypes of single nucleotide polymorphisms in the SCD and control groups: (a) rs6874185; (b) rs34554140; (c) rs6670279; * statistically significant differences between the groups, p<0.05

The frequencies of genotypes for rs34554140 significantly differed between the SCD cases and controls (p=0.005): the proportion of AA genotype carriers in the SCD group was significantly higher than that in the control group (p=0.002; OR=1.85; 95% CI 1.26–2.71); the proportion of AT genotype carriers in the SCD group was significantly lower than that in the control group (p=0.001; OR=0.53; 95% CI 0.36–0.78). When subdivided by gender or age, the differences between SCD cases and controls persisted in the subgroups of men, women, and <50-year-olds (p<0.05).

For the frequencies of rs6670279 genotypes, statistically significant differences were also found between the groups (p=0.005): the proportion of AA genotype carriers was significantly higher in the SCD group than that in control (p=0.005; OR=1.54; 95% CI 1.15–2.06); the proportion of AT genotype carriers in the SCD group was significantly less than that in the control group (p=0.047; OR=0.73; 95% CI 0.54–0.98). When subdivided by gender or age, the differences between SCD and control persisted in the subgroups of men, subjects >50 y.o., and men >50 y.o. (p<0.05).

In the control group, a statistically significant association of rs6670279 with waist circumference was found (p=0.047; ANOVA test). In carriers of the AA genotype, the average waist circumference was 95.5±1.4 cm, in carriers of the AT genotype — 89.5±1.4 cm, in carriers of the TT genotype — 99.1±8.9 cm. When groups were divided by gender, no significant association of polymorphism genotypes with waist circumference was found.

In the SCD group, we revealed a correlation between the rs6670279 polymorphism genotypes and the thickness of the right ventricular myocardium. In carriers of the AA genotype, the thickness of the right ventricular myocardium was 0.4 [0.3; 0.5] cm that was significantly less compared to carriers of other genotypes — 0.5 [0.4; 0.6] cm (p=0.013; Mann–Whitney test).

## Discussion

Single nucleotide polymorphisms rs34554140, rs6670279, and rs6874185 appear in the list of potential genetic markers of SCD we proposed following our genome-wide allelotyping of pooled human DNA [4]. To rule out false-positive results, the current scientific standards require molecular genetic studies to be verified using routine laboratory procedures. Another reason for the present research was that earlier [4] we used a limited number of DNA samples (200 men from the SCD group). In the current study, we aimed to expand the number of subjects by including both men and women, which is an important factor of data verification.

Previously, according to the available scientific literature, studies on the rs34554140, rs6670279, and rs6874185 polymorphisms were not reported in any Russian or foreign publications.

Single nucleotide polymorphism rs34554140 (g.36759697A>T) is located on chromosome 9. According to the dbSNP database [5], the frequency of the rare T allele in Caucasians is about 0.11. Our results show that the T allele frequency in the control group was also 0.11. The AA genotype of this polymorphism is associated with a high risk of SCD, and the AT genotype — with a low risk of SCD. Notably, these genotypes retain their association with SCD in the subgroups of both men and women, as well as in the subgroup of persons under 50 years of age. No associations of this polymorphism with any anthropometric, biochemical, or forensic data were found. Therefore, at this stage of the study, it is impossible to assess the contribution of this polymorphism to the development of SCD; however, it can be concluded that rs34554140 is indeed a new molecular genetic marker of SCD.

Single nucleotide polymorphism rs6670279 (g.110487907T>A) is located on chromosome 1. The frequency of the rare T allele in Caucasians is about 0.23 [6]. According to the present results, the frequency of this T allele in the control group was 0.22. The AA genotype of the polymorphism is associated with a high SCD risk, and the AT genotype is associated with a low risk of SCD. For the gender and age subgroups, the above association of the genotypes with SCD persists in the subgroups of men, individuals >50 y.o., and men >50 y.o. An association of the polymorphism genotypes with waist circumference in the control group was also revealed. In carriers of the AT genotype, with a low risk of SCD, the waist circumference was significantly less than that in carriers of the other two genotypes. As reported, the clinical significance of waist circumference depends on gender [7]; therefore, we analyzed the association of waist circumference with polymorphism genotypes separately for men and women. In this gender-specific analysis, the association was not confirmed. Therefore, with a high degree of probability, the association of polymorphism with waist circumference can be considered an accidental find that does not have any scientific significance.

An association of the rs6670279 polymorphism with the thickness of the right ventricular myocardium in the SCD group was also found: in carriers of the AA genotype, which is the SCD risk genotype, the thickness of the right ventricular myocardium was significantly less than that in carriers of the TT and AT genotypes. It is currently not possible to make any conclusion about the relationship between the myocardial wall thickness and the risk of SCD, since myocardial hypertrophy by itself may be a risk factor for SCD [8]. Thus, the obtained association does not fit into the system of existing SCD risk factors and, in all likelihood, is also an accidental finding and has no correlation with SCD. It can be concluded that the rs6670279 SNP may indeed be a new genetic marker of SCD, but its association with the thickness of the right ventricular myocardium requires additional testing in subjects with varying thickness of the right ventricular myocardium.

Single nucleotide polymorphism rs6874185 (g.176143664C>T) is located on chromosome 5. The frequency of the rare T allele in Caucasians is about 0.25 [9]. Our data show the frequency of this T allele in the control group as 0.19. According to this verification study, no association of rs6874185 polymorphism with SCD was confirmed, i.e. SNP rs6874185 is not a molecular genetic marker of SCD. This negative result once again confirms the need to verify the results obtained by molecular genetic methods with the help of confirmatory studies using routine techniques.

## Conclusion

The present results confirm that the single nucleotide polymorphisms rs34554140 and rs6670279 are associated with SCD and thus verify our earlier results obtained by genome-wide allelotyping. The AA genotypes of these two polymorphisms are associated with an increased risk of SCD, and the AT genotypes have a protective effect against SCD. No association between SCD and the rs6874185 SNP was revealed in this study.
